# Comprehensive Analysis of the Potential Prognostic Value of 11 Glycosylation-Related Genes in Head and Neck Squamous Cell Carcinoma and Their Correlation with PD-L1 Expression and Immune Infiltration

**DOI:** 10.1155/2022/2786680

**Published:** 2022-04-14

**Authors:** Langxiong Chen, Yuefu Ling, Hong Yang

**Affiliations:** ^1^Head and Neck Surgery, Affiliated Cancer Hospital and Institute of Guangzhou Medical University, Guangzhou, Guangdong Province, China; ^2^Otolaryngology & Head and Neck Surgery, Affiliated Hospital of Guilin Medical University, Guilin, Guangxi Zhuang Autonomous Region, China

## Abstract

**Background:**

Head and neck squamous cell carcinoma (HNSCC) is one of the worst and most common malignant tumors. This study is aimed at studying the complex interaction between glycosylation-related genes and HNSCC.

**Methods:**

The Cancer Genome Atlas (TCGA) contains gene expression profile data of HNSCC and normal tissues, as well as patient survival and clinical data. Combining five glycosylation-related gene sets, bioinformatics was used to analyze the expression of glycosylation-related genes in TCGA-HNSCC datasets and to identify prognostic risk markers, analyze their prognostic value, and the influence of glycosylation-related genes on the tumor immune microenvironment.

**Results:**

Gene expression profiles and corresponding clinical information of 499 cases of HNSCC and 44 cases of adjacent tissues were obtained. Using 11 glycosylation-related genes to construct a prognostic risk score, the Kaplan-Meier curve analysis found that the overall survival of the high-risk group was significantly different than that of the low-risk group (*P* < 0.001). ROC analysis was used to evaluate the prognostic efficacy of prognostic risk markers, and the results showed that the prognostic risk markers had good efficacy in predicting the prognosis of patients. We also found that there is a correlation between glycosylation-related genes, PD-L1, and immunocyte infiltration, and there is a dynamic effect between the change in the copy number of glycosylation-related genes and the number of tumor-infiltrating immune cells.

**Conclusions:**

Our research shows that glycosylation-related prognostic risk markers may be independent risk factors for the prognosis of HNSCC. We have found that there may be subtle links between glycosylation-related genes, PD-L1, and immunocyte infiltration, which has certain significance for exploring the occurrence and development of HNSCC and exploring the research of targeted therapy.

## 1. Introduction

Head and neck cancer is one of the main causes of global morbidity and mortality; among which more than 90% are squamous cell carcinomas, and most are derived from the stratified squamous mucosa of the lips, oral cavity, oropharynx, hypopharynx, and epithelial larynx [1]. Current studies have shown that the occurrence and development of head and neck squamous cell carcinoma (HNSCC) are related to smoking, long-term chewing of betel nuts, heavy drinking, and increasingly, high-risk human papillomavirus (HPV) infections [2, 3]. Although with the improvement of medical standards and the development of advanced medical equipment, an increasing number of patients can be diagnosed and treated at an early disease stage. As one of the most common malignant tumors, the 5-year overall survival (OS) period for patients with HNSCC is only approximately 50% [4]. Tumor biomarkers are considered markers that can assist in the diagnosis and understanding of the occurrence and development of malignant tumors and can predict and identify specific cancer types, improve clinical diagnosis and treatment, and provide predictive information about the response to treatment [5]. Therefore, it is of great significance to explore effective tumor markers and design new treatment strategies for HNSCC patients.

The research found that mechanisms related to abnormal glycosylation play an important role in promoting the occurrence and development of HNSCC [6]. The study also found that there are many cytokines and immunosuppressive cells related to tumor immune escape in the HNSCC microenvironment [7]. Programmed death ligand 1 (PD-L1) positive advanced and metastatic HNSCC patients are likely to be sensitive to immunotherapy, immune checkpoints such as programmed death 1 (PD-1) treatment have received widespread attention, and related inhibitory antibodies can improve the prognosis of patients with HNSCC [8].

Abnormal glycosylation is one of the unique characteristics of cancer cells. Specific glycan changes and abnormal glycosylation processes are crucial in tumorigenesis and metastasis [9]. Studies show that glycan structures, glycosylated proteins, and glycosylation enzymes have influence on different steps of the metastatic process, including epithelial-mesenchymal transition (EMT), migration, invasion/intravasation, and extravasation of tumor cells [10]. Studies have found that abnormal glycosylation plays a key role in promoting the occurrence and development of HNSCC. These changes promote the proliferation, invasion, and metastasis of HNSCC [11]. Therefore, identifying reliable prognostic markers is very important for selecting appropriate targeted therapy and improving the prognosis of HNSCC patients. In addition, the correlation between glycosylation-related genes and PD-L1, the expression of PD-L1 in HNSCC, and the abundance of immune infiltrating cells all need to be further studied. This study intends to analyze the transcriptome sequencing data, clinical data, and immune cell data of HNSCC, construct glycosylation-related genes as prognostic risk markers, and study its potential clinical application value. In addition, we also studied the infiltration of immune cells, the expression of PD-L1 in HNSCC, and the correlation with glycosylation-related genes.

## 2. Materials and Methods

### 2.1. TGCA Head and Neck Squamous Cell Carcinoma Gene Expression Profile and Clinical Data

The genome-wide expression profiles and clinical data of patients with HNSCC were extracted from The Cancer Genome Atlas (TCGA) dataset. Gene profile expression comparing HNSCC tissues (*n* = 499) and normal tissues (*n* = 44) and the corresponding clinical-related information were obtained.

### 2.2. Prognostic Glycosylation-Related Genes and Construction of New Predictive Risk Markers

Using gene set enrichment analysis, five glycosylation-related gene sets were obtained. The gene set files are from the Molecular Signatures Database and were in GMP format. We analyzed the expression differences of the glycosylation-related genomes in HNSCC tissues and adjacent tissues. *P* < 0.05 was considered to have a significant difference in gene expression, and the core gene signature was constructed. Through single-factor and multifactor Cox regression analysis based on core genes, prognostic risk indicators were selected and prognostic risk indicators established.

### 2.3. Statistical Methods and Survival Analysis

R language was used to screen for glycosylation-related genes with differential expression and to analyze the correlation between potential prognostic risk markers and other clinical variables. The R language loaded with the limma (linear model of microarray data) package was necessary for the statistical analysis of this study. Each patient was assigned a unique risk score based on the linear combination of gene expression levels: Risk score = expression value of gene 1 × *β*1 + expression value of gene 2 × *β* 2 + ⋯+expression value of gene *n* × *β* *n* expression.

Using the risk score value to obtain the median as a cut-off value, 499 patients with HNSCC were divided into high- and low-risk groups. The Kaplan-Meier curve was used for survival analysis, and ROC analysis was used to predict the prognostic efficacy of the labels. Univariate and multivariate Cox regression analysis was used to analyze the correlation between predictive signatures of glycosylation-related genes and other clinical variables. A *P* value less than 0.05 was considered statistically significant.

cBioPortalc is an online analysis database that provides visualization tools for research and analysis of cancer genetic data and uses molecular data obtained from cancer tissue and cytology to understand heredity, epigenetics, gene expression, and proteomics. STRING version 11.0 is an online tool that can modify and integrate information from many sources to analyze interactions between glycosylation-related genes.

## 3. Results

### 3.1. Glycosylation-Related Gene Set

**Figure 1:**
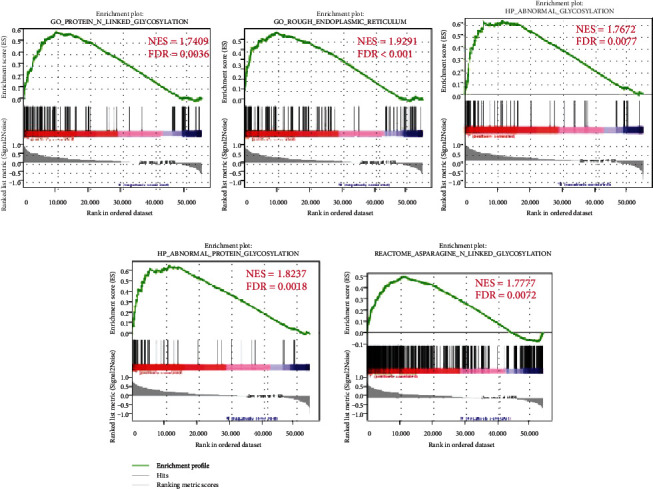
GSEA analysis of the enrichment of five glycosylation-related gene sets between HNSCC and normal tissues.

The datasets included gene profiles of throat squamous cell carcinoma and oral squamous cell carcinoma. All information is derived from the Cancer Genome Atlas database (https://portal.gdc.cancer.gov/repository). We obtained and screened glycosylation-related gene sets on the GSEA website (https://www.gsea-msigdb.org/gsea/login.jsp), and we used five different gene sets (GO_PROTEIN_N_LINKED_GLYCOSYLATION, GO_ROUGH_ENDOPLASMIC_RETICULUM, HP_ABNORMAL_GLYCOSYLATION, HP_ABNORMAL_PROTEIN_GLYCOSYLATION, and REACTOME_ASPARAGINE_N_LINKED_GLYCOSYLATION) to explore whether these five glycosylation-related gene sets are different between the paracancer sample and the tumor sample. We found that the abovementioned gene set in HNSCC was significantly different than that of the adjacent tissues and tumor tissues (FDRs were 0.003, <0.001, 0.007, 0.002, and 0.007, respectively, Figure 1). Next, *P* value < 0.05 considered that the expression of glycosylation-related genes in HNSCC and normal tissues was significantly different, the glycosylation gene set was screened, and the core genes are thus obtained.  Table 1 shows genes with significant differences in expression in tumor tissues.

**Figure 2:**
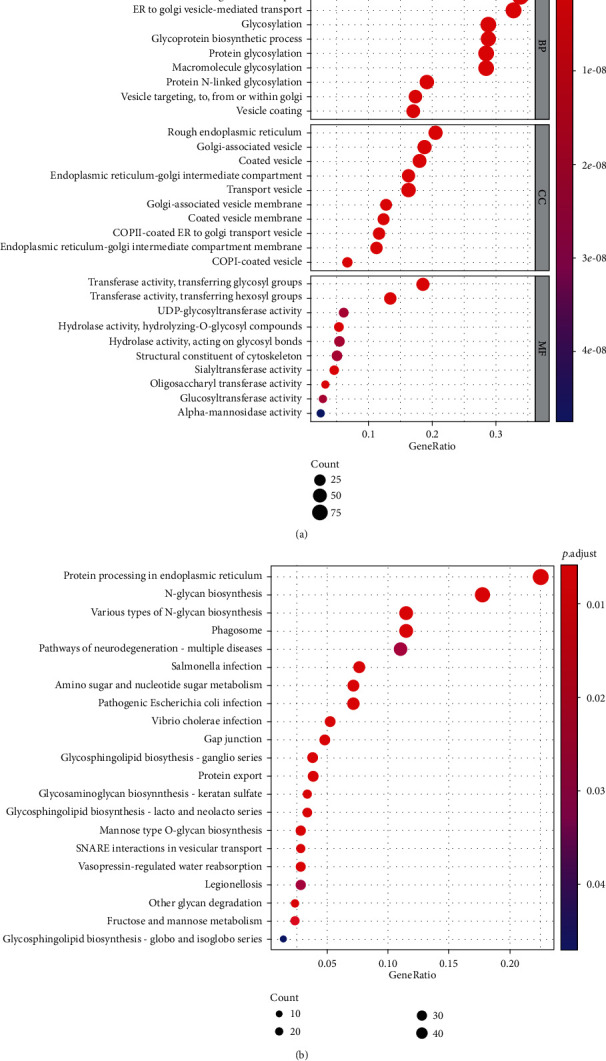
Functional enrichment analysis of core genes: (a) GO enrichment analysis and (b) KEGG enrichment analysis.

To verify whether the core genes were related to glycosylation, we used GO analysis and KEGG pathway enrichment analysis for more in-depth analysis.  Figure 2 shows that the most enriched biological process (BP) is related to a variety of glycosylation processes, and molecular functions (MF) are related to multiple glycosylation pathways including glycosyl transfer, UDP-glycosyltransferase activity, O-Glycosyl compounds, and other related; KEGG pathway enrichment analysis involves protein processing in the endoplasmic reticulum, N-glycan biosynthesis, and glycosphingolipid biosynthesis, suggesting that the core genes screened are related to glycosylation.

### 3.2. Construction of Prognostic Risk Markers Based on Core Genes

To define the OS data of HNSCC patients in association with core genes, single-factor Cox regression analysis was used to screen glycosylation genes related to OS of patients. Multivariate Cox regression analysis further verified the correlation between glycosylation-related core genes and patient OS. After the analysis, prognostic risk markers related to OS were screened, and the result was a prognostic risk marker composed of OS-related glycosylation-related genes, namely, PSMC1, NAGK, AREG, DDOST, ATP6V1E1, KDELR1, PLOD2, TMED10, ALG5, ARF3, and OST4 (Table 2).

**Figure 3:**
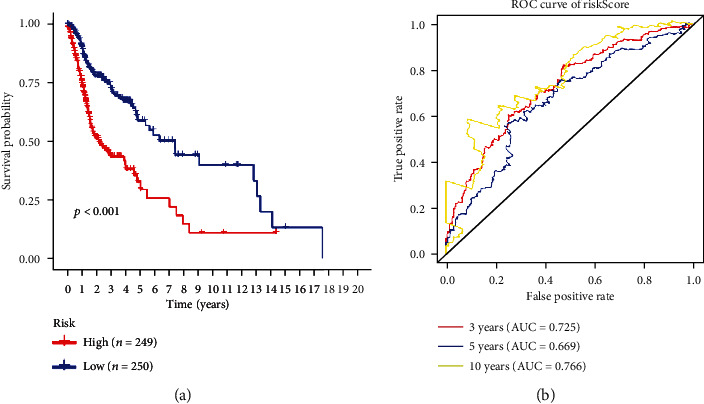
Survival analysis of prognostic risk markers: (a) Kaplan-Meier curve analysis of OS between high-risk groups and low-risk groups and (b) ROC curve analysis of diagnostic power.

**Figure 4:**
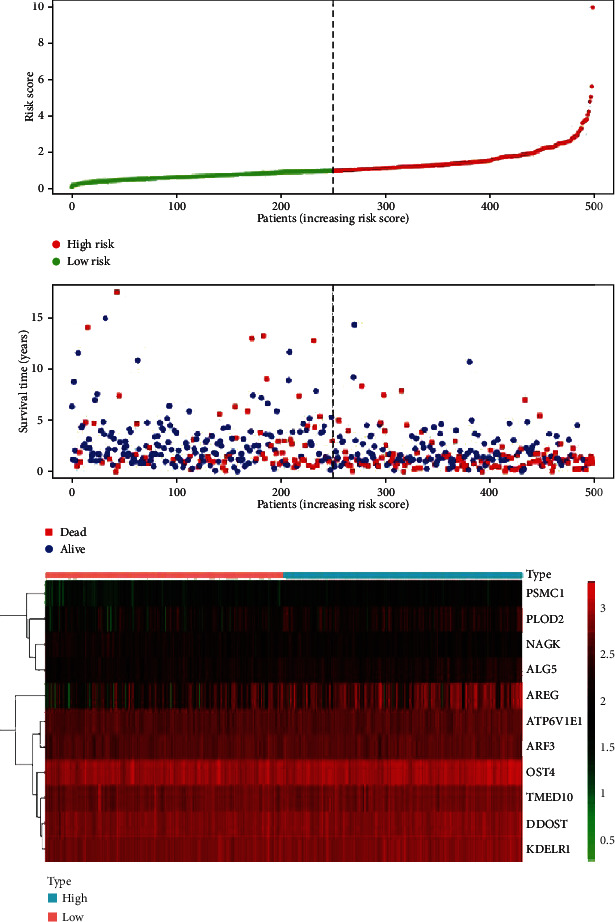
Risk curve analysis of survival status of high- and low-risk groups.

The regression coefficient (*β*) of each prognostic risk index was calculated by multivariate Cox analysis. The unique risk score of these patients is obtained based on the regression coefficient and the expression level, where the risk score value (Risk score) = (PSMC1 expression × 0.549384) + (NAGK expression×−0.35266) + (AREG expression × 0.133426) + (DDOST expression×−0.31938) + (ATP6V1E1 expression × 0.424889) + (PLOD2 expression × 0.149545) + (TMED10 expression×−0.34939) + (ALG5 expression × 0.323277) + (ARF3 expression × 0.617688) + (OST4 expression × 0.407259) + (KDELR1 expression × 0.37953). Based on unique risk score, HNSCC patients were divided into a high-risk group and a low-risk group according to the median value of the risk score, with a risk score greater than the median being classified as a high-risk group (*n* = 249) and a risk score less than the median being classified as low-risk risk group (*n* = 250). The Kaplan-Meier (KM) survival curve was used to assess the prognostic difference between the two groups. The results showed that the OS of the high-risk group was significantly different from that of the low-risk group (*P* value < 0.001) (Figure 3(a)). To test the effectiveness of 11-gene glycosylation-related prognostic risk markers in predicting OS in patients with HNSCC at 3, 5, and 10 years in the diagnosis of HNSCC, we used receiver operating characteristic curve (ROC) analysis that further verified its diagnostic efficacy. The results showed that the respective area under the curve (AUC) was 0.725, 0.669, and 0.766, respectively (Figure 3(b)), indicating that prognostic risk markers perform well in predicting OS in patients with HNSCC. Based on the risk score, we drew a risk curve (Figure 4) and found that the higher the risk score, the shorter the patient's survival time.

### 3.3. Analysis of the Expression and Correlation of 11 Glycosylation-Related Genes in Head and Neck Squamous Cell Carcinoma

**Figure 5:**
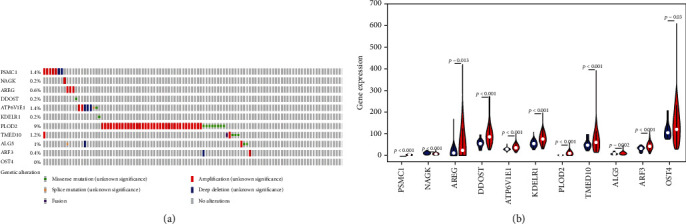
Analysis of the expression of 11 glycosylation-related genes in HNSCC: (a) gene mutations in HNSCC and (b) gene expression differences in HNSCC and adjacent tissues.

In the cBioPortal online database, we analyzed the mutations of 11 glycosylation-related genes in HNSCC through clinical samples. The results showed that 78 patients (15.6%) had genetic mutations. Among them, the rate of mutation of the PLOD2 gene was 9%, including 37 cases of amplification and 8 cases of missense mutations. PSMC1 and ATP6V1E1 gene mutations accounted for 1.4%, TMED10 gene mutations accounted for 1.2%, and ALG5 gene mutations accounted for 1%; in addition, NAGK, AREG, DDOST, KDELR1, ARF3, and OST4 gene mutation rates were all lower than 1% (Figure 5(a)).

**Figure 6:**
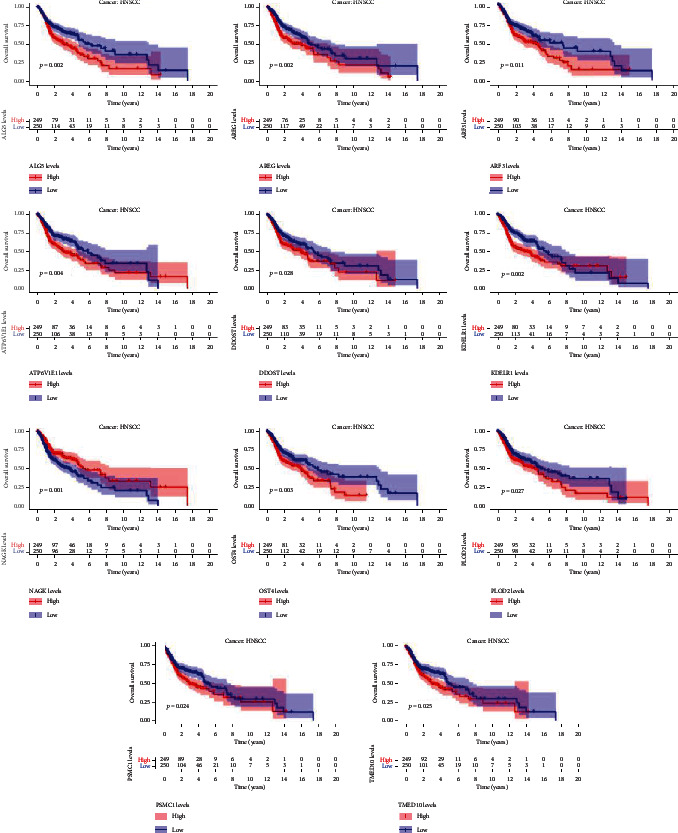
Based on the expression of each glycosylation-related gene in HNSCC, Kaplan-Meier curve analysis predicted the overall survival (OS) of HNSCC patients stratified into high- and low-expression groups of the indicated genes.

We further analyzed the differential expression of PSMC1, NAGK, AREG, DDOST, ATP6V1E1, KDELR1, PLOD2, TMED10, ALG5, ARF3, and OST4 in HNSCC and normal head and neck tissues and found that compared with normal tissues, there are 11 glycosyl groups in HNSCC tissues. The expression of metabolism-related genes is significantly different (*P* < 0.001 for PSMC1, NAGK, DDOST, ATP6V1E1, KDELR1, PLOD2, TMED10, and ARF3; *P* = 0.013 for AREG; *P* = 0.002 for ALG5; *P* = 0.03 for OST4) (Figure 5(b)). We classified the expression of each gene in tumor samples from HNSCC patients and then divided these patients into two subgroups based on the median expression value, namely, the high-expression group and the low-expression group. The Kaplan-Meier curve was used to verify whether the high or low expression of each gene was associated with the OS in patients with HNSCC. PSMC1, NAGK, AREG, DDOST, ATP6V1E1, KDELR1, PLOD2, TMED10, ALG5, ARF3, and OST4 were found to be related to a poor prognosis of HNSCC (*P* = 0.004, <0.001, 0.027, 0.035, 0.030, and 0.009, respectively) (Figure 6). For PSMC1, NAGK, AREG, DDOST, ATP6V1E1, KDELR1, PLOD2, TMED10, ALG5, ARF3, and OST4, their role as independent biomarkers of prognosis needs to be further verified. Thus, we used ROC curve analysis to further confirm their prognostic effects (shown in Table 3), but the AUC values of the 11 glycosylation-related genes that predicted OS in HNSCC patients were below 0.725, 0.669, and 0.766, respectively, indicating that they were predictors of poor prognosis.

**Figure 7:**
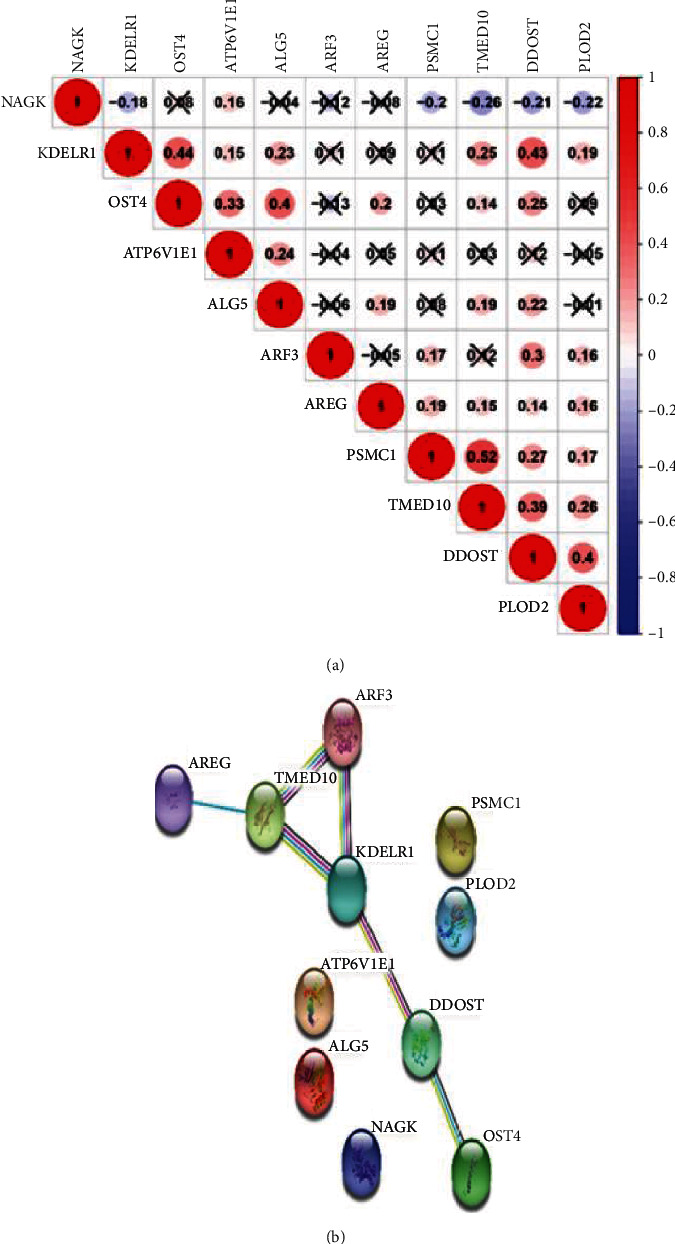
Gene correlation analysis: (a) Pearson correlation coefficient and (b) PPI network.

The Pearson correlation coefficient was used to test the strength of the coexpression of glycosylation-related genes. As shown in Figure 7, we found that 11 gene pairs showed a coexpression relationship. Among these gene pairs, the TMED10-PSMC1 pair has the strongest correlation (0.52); while ATP6V1E1 and ALG5 showed the lowest correlation with other glycosylation-related genes, and NAGK was negatively related to the other glycosylation-related genes. We used the online tool STRING version 11.0 to construct a protein-protein interaction (PPI) network. We found that the genes TMED10, ARF3, and KDELR1 were closely related and seemed to be associated with other genes.

### 3.4. Correlation between Prognostic Risk Markers and Clinical Characteristics

**Figure 8:**
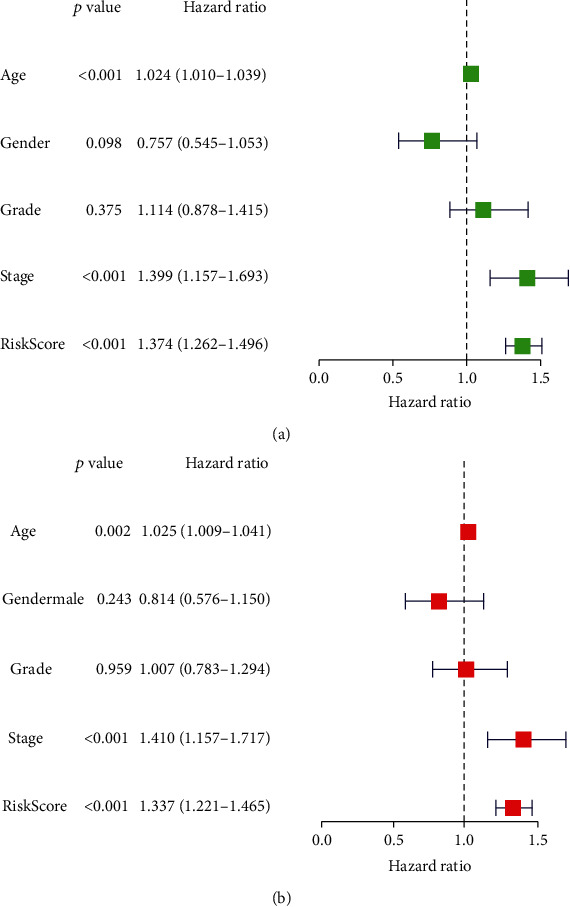
Univariate and multivariate Cox analysis to analyze the relationship between risk score and clinical characteristics: (a) univariate Cox analysis and (b) multivariate Cox analysis.

We then evaluated whether the glycosylation-related prognostic risk markers composed of 11 oncogenes were related to clinical parameters including sex, age, grade, and clinical stage of patients with HNSCC. We used clinical parameters as covariates, analyzed the entire data set using univariate and multivariate Cox regression, and analyzed the correlation between prognostic markers and covariates. The results showed that in univariate Cox regression analysis, age, tumor clinical stage, and risk score were significantly correlated with OS, while sex and tumor grade were not. After further analysis by multivariate Cox regression, the results showed that the risk score was significantly correlated with the OS of patients with HNSCC (*P* < 0.001, 95% CI 1.221-1.465, HR = 1.337). The results of univariate and multivariate Cox regression analysis showed that prognostic risk signature composed of 11 glycosylation-related genes can predict the prognosis of patients and can be independent of clinical characteristics. In summary, prognostic risk markers can be applied to predict OS in patients with HNSCC (Figure 8).

**Figure 9:**
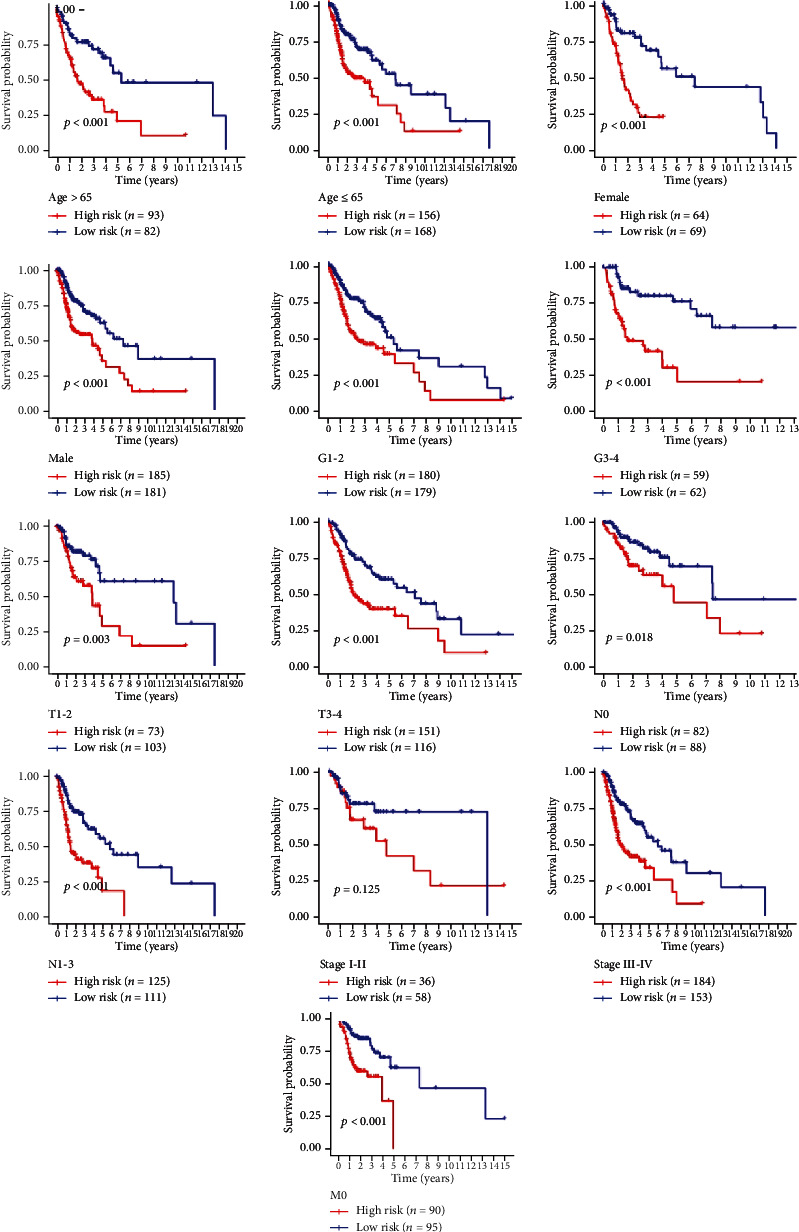
Kaplan-Meier curve analysis of the survival relationship between risk score and clinical characteristics.

We carried out a stratified analysis of each clinical feature and further evaluated the correlation between the risk score and the clinical feature. The result is shown in Figure 9. We found that when patients were stratified according to age, patients older than 65 years old have high-risk subgroups (*n* = 93) and low-risk subgroups (*n* = 82); while patients younger than 65 years old have high risk subgroup (*n* = 156) and low-risk subgroup (*n* = 168), there was a significant difference in OS between the two subgroups (*P* < 0.001). Patients were stratified by sex; for the females, 64 were in the high-risk subgroup and 69 were in the low-risk subgroup, while for males, there were 185 cases in the high-risk subgroup and 181 cases in the low-risk subgroup. The analysis showed that there were significant differences in OS rates between the two subgroups (*P* < 0.001).

All patients were stratified according to tumor grade (G) and were divided into two subgroups: GI/GII (*n* = 359) and GIII/GIV (*n* = 121). According to the risk score, there were 180 cases in the high-risk subgroup and 179 cases in the low-risk subgroup for GI/GII. We found a significant difference in OS between the two subgroups (*P* < 0.001). The same results were also observed in the two subgroups of patients with GIII/GIV tumors (*P* = 0.003). Patients were divided into T1 and T2 groups (*n* = 177) and T3 and T4 groups (*n* = 266) based on the T stage of the tumor. The survival rate of the low-risk subgroup in the T1 and T2 groups was significantly different from that of the high-risk subgroup (*P* = 0.003), and similar survival rates of the high- and low-risk subgroups in the T3 and T4 groups were also found (*P* < 0.001). Depending on the presence or absence of lymph node metastases, these patients were divided into two groups. We found that the survival rates of the low-risk and high-risk subgroups were significantly different between the two groups (*P* = 0.018 and *P* < 0.001, respectively). We divided patients into stage I and II groups (*n* = 94) based on the clinical stage of the tumor, which was high-risk subgroups (*n* = 36) and low-risk subgroups (*n* = 58). Stages III and IV were grouped together (*n* = 337), for the high-risk subgroup (*n* = 184) and low-risk subgroup (*n* = 153). In tumor stage III and IV groups, the survival rate of patients in the high-risk subgroup was significantly different from that of patients in the low-risk subgroup (*P* < 0.001). In the tumor clinical stages I and II groups, there was no significant difference in the survival rate of patients in the two subgroups (*P* = 0.125). Since there was only 1 patient with distant metastasis, we could only stratify patients without distant metastasis to obtain a high-risk subgroup and a low-risk subgroup. The results suggested that there was a significant difference in survival rate between the two groups (*P* < 0.001).

### 3.5. The Relationship between PD-L1 and Glycosylation-Related Genes

**Figure 10:**
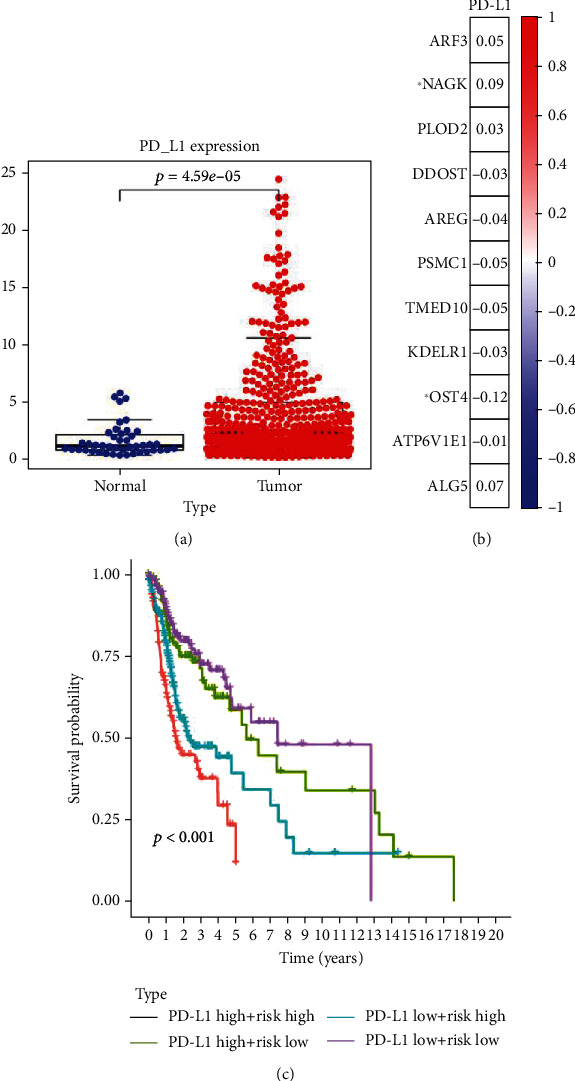
(a) Expression of PD-L1 in HNSCC tissue and normal tissue. (b) Correlation between PD-L1 and 11 glycosylation-related genes. (c) A significant difference in overall survival between 4 subgroups.

We assess the difference in expression of PD-L1 in tumor tissue and normal tissues of HNSCC patients (Figure 10(a)). Compared to normal tissues, the expression of PD-L1 in HNSCC tissue increased significantly. We analyzed the relevance of PD-L1 and 11 glycosylation-related genes (Figure 10(b)). We have found that the expression of PD-L1 is significantly positively correlated with the expression of NAGK, and the expression of PD-L1 is significantly negatively correlated with the expression of OST4. HNSCC patients were divided into high expression groups and low expression groups according to the expression level of PD-L1, and the two groups were divided into four subgroups according to the risk score. We found that there is a significant difference in overall survival between 4 subgroups (Figure 10(c)).

### 3.6. The Effects of Genetic Changes in Glycosylation-Related Prognostic Risk Markers on Immune Cell Infiltration

**Figure 11:**
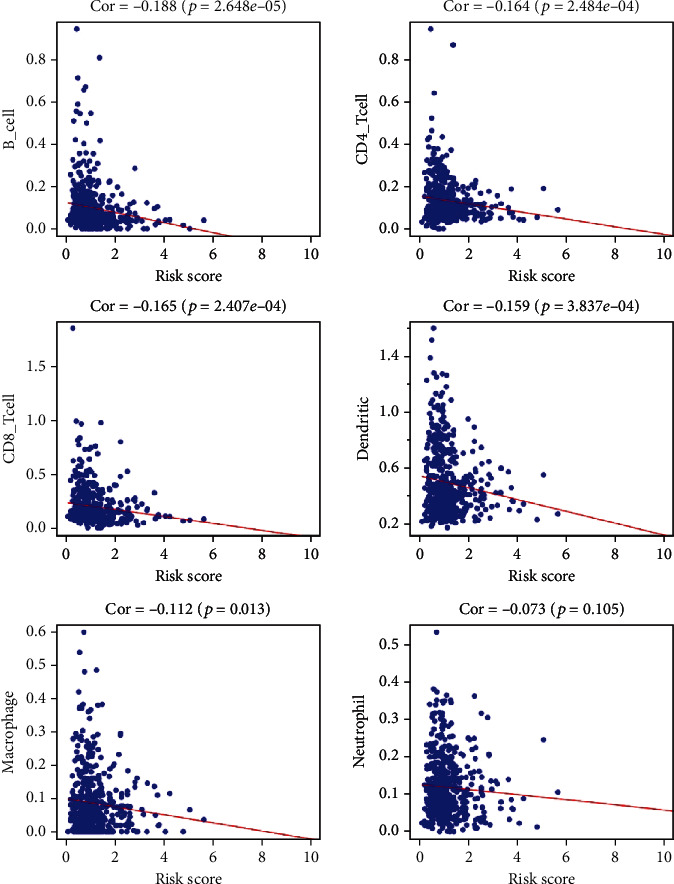
The relationship between risk score and immune cell infiltration level.

We analyzed the relationship between prognostic risk markers and the level of infiltration of six immune cell types (Figure 11). The results showed that there was a significant negative correlation between the risk score and the level of infiltration of B cells, CD4+ T cells, CD8+ T cells, dendritic cells, and neutrophils. There was no significant correlation between the risk score and the level of macrophage infiltration. The results confirmed that signatures based on glycosylation-related genes were related to the TIME of HNSCC.

**Figure 12:**
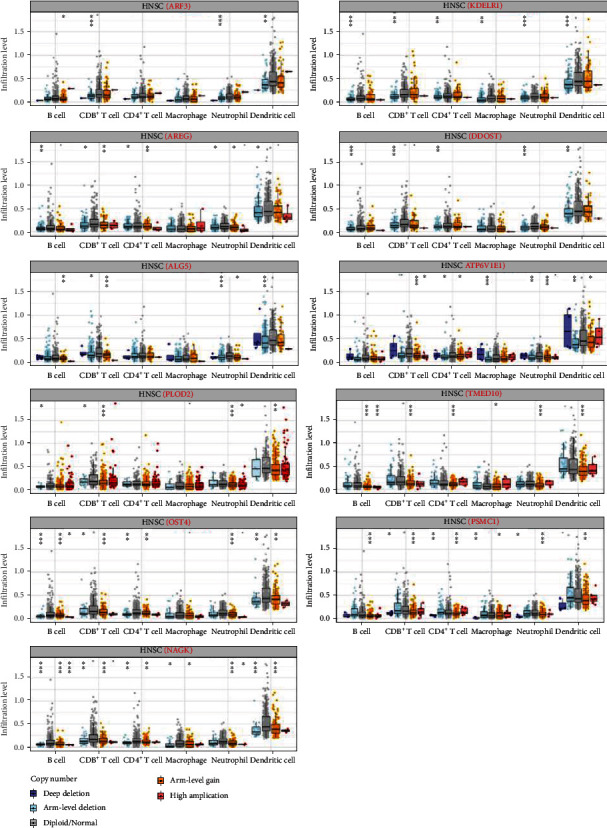
The effects of somatic copy number alteration (CNA) on the expression of glycosylation-related genes on immune cell infiltration. ∗*P* < 0.05, ∗∗*P* < 0.01, and ∗∗∗*P* < 0.001.

The effects of somatic copy number alteration (CNA) based on glycosylation-related genes on immune cell infiltration were further analyzed. The CNA of identified glycosylation-related genes significantly influenced the infiltration of B cells, CD4+ T cells, CD8+ T cells, neutrophils, macrophages, and dendritic cells in HNSCC. These results indicated that glycosylation-related genes had a key regulatory effect on the TIME in HNSCC patients (Figure 12).

## 4. Discussion

The occurrence, development, and invasion of tumors are accompanied by changes in glycosylation of related glycoproteins. The changes in the structure of carbohydrates on the surface of cancer cells play an important role in the course of cancer [12]. The process of glycosylation affects the complexity of the regulation of protein function and promotes tumor proliferation, invasion, and angiogenesis [13, 14]. Abnormal glycosylation mediated by glycoproteins such as E-cadherin, PD-1/PD-L1, EGFR, and CD44 can have a vital impact on the epithelial-mesenchymal transition and immune escape of HNSCC [15]. However, the specific mechanism of abnormal protein glycosylation on the occurrence and development of HNSCC has not been fully elucidated. We constructed 11 glycosylation-related prognostic risk markers composed of PSMC1, NAGK, AREG, DDOST, ATP6V1E1, KDELR1, PLOD2, TMED10, ALG5, ARF3, and OST4 from the glycosylation-related gene set, and calculated risk scores perform well in predicting the prognosis of HNSCC patients, indicating that the prognostic risk markers composed of glycosylation-related genes can be used as predictive biomarkers for HNSCC to predict the prognosis of HNSCC. Subsequent univariate and multivariate Cox proportional hazards regression analysis showed that the risk score was significantly related to the patient's OS, indicating that the risk score can be independent of other clinical characteristics. This study constructed prognostic risk markers related to glycosylation as a prognostic marker for HNSCC patients, providing new ideas and molecular targets for HNSCC research and individualized treatment. Our results indicate that predictive features composed of glycosylation-related genes have shown potential in predicting the prognosis of HNSCC patients and personalized treatment.

Abnormal glycan structure and glycosylation process may be an important factor in the complex process of immune escape of tumor cells [16]. The protein stability of PD-L1 may be affected by ubiquitination, phosphorylation, and glycosylation, while altering its protein-protein interaction [17]. Studies have shown that N-glycosylation plays a key role in maintaining the protein structure stability of PD-1, which affects antitumor immune responses, while inhibition of Fut8 affects cell surface PD-1 expression, T cell activation be enhanced, thereby affecting the occurrence and development of tumors [18]. We found that the expression of PD-L1 in HNSCC tissue increased significantly. Further, the results show that the expression of PD-L1 is significantly positively correlated with the expression of NAGK, and the expression of PD-L1 is significantly negatively correlated with the expression of OST4. HNSCC patients were divided into high expression groups and low expression groups according to the expression level of PD-L1, and the two groups were divided into four subgroups according to the risk score. We found that there is a significant difference in overall survival between 4 subgroups. The results show that the PD-L1 may have close contact with the glycosylation-related genes, and this connection also affects the overall survival of HNSCC patients to a certain extent.

Immune-related cells are considered an indispensable key part of the innate and adaptive immune system, such as B cells, T cells, dendritic cells (DCs), and natural killer (NK) cells. During tumorigenesis, abnormal glycosylation processes can directly or indirectly affect the immune response of tumor cells, thereby regulating tumor progression [19]. Exploring the role of protein glycosylation in the structure and function of immune cells, immunoglobulins and immune factors may help reveal the molecular mechanisms of glycosylation in tumor immunity and may suggest new treatments to inhibit tumor recurrence and metastasis. At present, the effect of glycosylation-related genes on immune cell infiltration in the tumor immune microenvironment is still unclear. In this study, the results of immune cell infiltration analysis based on 11 glycosylation-related genes showed that the risk score was significantly correlated with the infiltration levels of B cells, CD4 + T cells, CD8 + T cells, dendritic cells, and neutrophils related.

The small sample size in the validation data set underlines the limitations of this study and suggests there may be selection bias in the results. To verify the reliability of the conclusions, more prospective clinical trials are necessary. In addition, our study only explored the potential correlation of glycosylation-related genes, PD-L1 and immune infiltration in the occurrence and development of HNSCC, more detailed studies are needed to analyze the specific mechanisms of glycosylation-related genes in promoting HNSCC progression. It is necessary to further study how glycosylation-related genes, PD-L1 and immune infiltration, jointly regulate the occurrence and development of tumors. It provides clinicians with more reliable diagnostic markers, provides an important basis for finding potential targets for HNSCC treatment, and is expected to provide precise and personalized treatment strategies for HNSCC patients.

## 5. Conclusions

Our study identified an 11 glycosylation-related gene signature of prognostic risk markers, namely, PSMC1, NAGK, AREG, DDOST, ATP6V1E1, KDELR1, PLOD2, TMED10, ALG5, ARF3, and OST4. These markers can predict the prognosis of patients with HNSCC, indicating that glycosylation-related genes can be used as prognostic risk markers for HNSCC. We analyzed and evaluated the correlation between 11 glycosylation-related genes and PD-L1, as well as the potential regulatory mechanisms. The risk score of glycosylation-related genes is significantly related to the level of immune cell infiltration in HNSCC patients, and glycosylation-related genes may be involved in the regulation of the immune microenvironment of HNSCC. Therefore, identifying glycosylation-related genes and molecular pathways that affect tumor immune response and further study into their regulatory mechanisms may provide potential molecular targets for improving the sensitivity of HNSCC to targeted immunotherapy.

## Figures and Tables

**Table 1:** The core genes were selected from 5 glycosylation-related gene sets.

*n* = 282
ALG10B, NAPA, CTSZ, TMED3, CYBB, F8, GLUL, SYVN1, TUBB1, DYNC1LI2, RAB1A, TRAPPC6B, CDKAL1, NAT8L, LHB, ALG6, MT3, DYNC1I2, CAPZA1, CD55, FOLR1, GMPPA, LMAN2, NCF1, SEC22A, TMEM258, COPG1, MGAT4A, RPS23, EPHA5, EDEM3, TRAPPC4, DERL1, TUBA1C, ATP6V1A, FUT3, TUBB6, DPAGT1, PCSK9, UGGT1, TUBA3D, ST8SIA5, RPS28, TMEM97, PSMC1, NAGK, TP63, ST8SIA2, ST8SIA4, UGGT2, COPE, SEC22C, STT3B, ALG12, OSTC, AREG, SFTPA2, YKT6, TRAPPC2L, COG4, TMED9, TUSC3, ST6GALNAC2, NGLY1, SEC24C, APP, COPB2, GMDS, FKRP, SEC31A, ALG2, SLC7A11, SEC62, LMAN1L, KDELR2, DYNC1H1, HSPD1, SEC63, ST8SIA6, DERL2, FKTN, SEC61A2, GOSR2, PPP6R1, KDELR3, ST6GALNAC1, ST3GAL2, SPPL3, MACO1, MGAT5, UBC, GOSR1, NAPG, RNF103, SEL1L, DPM1, VCP, BGLAP, SEC23IP, AMDHD2, EDEM1, PKM, COPZ1, SPTBN2, MGAT5B, ST8SIA1, ALG1, EDEM2, PGM3, SEC23A, MIA2, CCDC115, STX5, SRP9, DDOST, MOGS, MGAT4B, B4GALT1, CNIH1, RAB1B, TGFA, CALR, CSNK1D, SLC35A2, TRIM13, ST6GALNAC5, SEC61G, USO1, MLEC, DERL3, SPTBN4, MGAT2, TMEM165, ANK3, PLOD3, LIN28A, ACTR1A, ST6GALNAC6, NANP, PLOD1, GLB1, GANAB, SERPINA1, B4GALT4, MAN1B1, ANKRD28, PGM1, PI4KB, SLC35C1, COL7A1, ATP6V1E1, SEC24D, UBE2J1, CTSC, ALG13, ALG10, RPL4, CHST8, TUBA3E, COG1, NUDT14, KRTCAP2, DCTN5, DPM2, SEC16A, TRAPPC3, GNPNAT1, PDIA3, CNIH2, STT3A, ST3GAL4, RPN2, COPA, GNRH1, CANX, ARSB, MGAT1, FUOM, TRAPPC1, TMED2, GNE, RP9, TRAPPC2, PRKCSH, NSF, COPB1, ARL6IP1, KDELR1, ALG3, RENBP, B4GALT2, GMPPB, LMAN1, RPN1, PLOD2, ATP6V0A2, UBXN1, F12, ST3GAL, BET1, PPP6R3, STAU1, BET1L, MYOC, LRAT, HM13, SEC61B, FPGT, DAD1, RAD23B, B4GALT7, RFT1, LRPAP1, SEC16B, NUS1, TMED10, FUT8, ALG5, ST3GAL1, ARCN1, COG2, ARF3, LMAN2L, ALG8, SRPRB, CNIH3, OST4, PREB, TUBA4A, CTSA, NEU3, ADCYAP1R1, CAPZB, SAR1B, PMM2, MAN2A1, ENGASE, MAN1C1, GBF1, NEU4, KCNJ2, TUBB3, ENTPD5, PSEN1, NUCB1, OS9, B4GALT3, COPZ2, SCGB1A1, ANK2, TMEM199, CAD, TUBA1B, GORASP1, NPL, BAIAP2, TUBAL3, GFPT2, ARF1, ARFGAP1, DCTN1, TMCC1, RNF139, MANEA, PTGDS, NEU1, NAPB, GRIA1, TFG, RANGRF, SCFD1, DOLK, SLC17A5, SPTA1, ARF5, CKAP4, CA4, ASGR1, SEC61A1, DOLPP1, ARFGAP3, B4GALT6

**Table 2:** Prognostic risk markers are screened from core genes based on bioinformatics analysis.

Prognostic risk markers					
Gene	*P* value	HR	HR.95L	HR.95H	Coeff (*β*)
PSMC1	1.27E-09	1.7435	1.2496	2.4327	0.549384
NAGK	1.92E-06	0.7046	0.5359	0.9264	-0.35266
AREG	0.013	1.1569	1.0662	1.2553	0.133426
DDOST	5.78E-16	1.3907	1.0065	1.9216	-0.31938
ATP6V1E1	1.44E-08	1.6330	1.1599	2.2992	0.424889
PLOD2	5.92E-16	1.2171	1.0527	1.4070	0.149545
TMED10	3.12E-05	1.3489	1.0248	1.7756	-0.34939
ALG5	0.002	1.7314	1.2715	2.3578	0.323277
ARF3	2.26E-10	1.4682	1.0263	2.1005	0.617688
OST4	0.030	1.5341	1.17272	2.0070	0.407259
KDELR1	8.27E-14	1.7969	1.2700	2.5423	0.37953

**Table 3:** ROC curve analysis of the prognostic diagnostic power of 11 genes.

ROC curve analysis of the prognostic diagnostic power of 11 genes			
Gene	(AUC)		
	3 years	5 years	10 years
PSMC1	0.629	0.559	0.526
NAGK	0.420	0.433	0.448
AREG	0.609	0.538	0.581
DDOST	0.571	0.601	0.523
ATP6V1E1	0.602	0.591	0.628
PLOD2	0.564	0.570	0.656
TMED10	0.595	0.541	0.501
ALG5	0.611	0.582	0.654
ARF3	0.579	0.564	0.630
OST4	0.570	0.592	0.668
KDELR1	0.596	0.579	0.481

## Data Availability

All the data and information used in the article are from public websites, including TCGA (https://portal.gdc.cancer.gov/repository), GSEA (https://www.gsea-msigdb.org/gsea/login.jsp), and the Molecular Signatures Database (https://www.gsea-msigdb.org/gsea/msigdb/index.jsp). Data sharing is not applicable to this article.
